# Development of a tool to enhance street cleaning service efficiency: The case study of Porto city, Portugal

**DOI:** 10.1177/0734242X251387004

**Published:** 2025-11-11

**Authors:** Maria Guedes, José C M Pires, Fernando G Martins, Carolina S Lucas, Hélder Claro, Manuel Fernando R Pereira, Joana M Dias

**Affiliations:** 1 Laboratory for Process Engineering, Environment, Biotechnology and Energy (LEPABE), Faculty of Engineering, University of Porto, Porto, Portugal; 2 EMAP-Porto Ambiente – Environment Company of the Municipality of Porto, Porto, Portugal; 3 Department of Mechanical Engineering, Faculty of Engineering, University of Porto, Porto, Portugal; 4 Associate Laboratory in Chemical Engineering (ALiCE), Faculty of Engineering, University of Porto, Porto, Portugal; 5 Department of Chemical and Biological Engineering, Faculty of Engineering, University of Porto, Porto, Portugal; 6 Laboratory of Separation and Reaction Engineering–Laboratory of Catalysis and Materials (LSRE-LCM), Faculty of Engineering, University of Porto, Porto, Portugal

**Keywords:** Cleaning indicators, cleanliness level, street sweeping, waste management, waste quantification, population survey

## Abstract

Urban cleanliness plays a crucial role in public health, environmental quality and citizens well-being, also affecting cities resilience. However, assessing street cleaning performance objectively, to improve services and citizen’s health, remains a challenge. The present study was devoted to create a robust methodology to determine urban cleaning performance with Porto Municipality as reference. Streets were analysed before the cleaning operations and the disposed waste was characterised by typology and size as well as by the zone where it’s found. The study considered 11 waste categories and 9 city areas together with developed indicators. Waste quantification was conducted in 52 streets, with 783 samples and 116,780 m^2^ monitored. Commercial/touristic and riverside areas were the cleanest, with cleanliness levels of 85% and 86%, respectively. Residential areas were the less clean, with cleanliness levels of 47% (social housing) and 54% (multi-family buildings), respectively. In residential and school areas, animal droppings were the most found category, corresponding to the highest annoyance level for the population, greatly affecting the streets’ cleanliness index. For Porto city, the cleanliness level obtained was 61%. According to the results obtained from 3,269 people surveyed, the cleaning status of Porto city has a score of 3.37 out of 5. Since the survey was conducted in the ‘lowest level of cleaning’, the results may be classified as good. The findings highlight the need for targeted cleaning strategies in lower-performing areas and demonstrate applicability for systematic urban cleanliness assessment. Future adaptations and optimisations could enable its use in broader contexts, supporting data-driven policymaking and improved urban management.

## Introduction

The proper cleaning of public space represents an important factor to the population, and it is a permanent challenge for municipal management services. The use of public spaces creates a variable and progressive dirtiness generation, always associated with unpredictable factors that hamper its cleanliness, particularly in places with a high touristic influx, such as Porto city. The generated dirtiness negatively impacts the environment, economy and tourism. Therefore, municipal services need to adopt several approaches to improve cleaning operations and, consequently, enhance street cleanliness (Alfarrarjeh et al., 2018; Lloyd et al., 2018). Therefore, it is essential to develop tools and mechanisms to monitor and control the quality of cleaning services to establish a high healthiness level in public spaces. Considering the variety of areas and activities around the city, it is highly complex to measure cleanliness and find reliable variables and, consequently, indicators to reflect its condition. On the other hand, it is crucial to find automatic procedures and tools to monitor street cleanliness so that evaluation errors are minimised and the control of operations is quicker and more practical. In addition, it is also important to evaluate whether the cleaning services are being executed according to established standards (Lopez et al., 2017).

Typically, the quality of these services must be monitored by considering performance indicators (Simoes and Marques, 2012). Unfortunately, in Portugal, there is little information on indicators developed to assess the streets’ cleanliness level. Around Europe, few research studies were conducted to develop methodologies to determine urban cleaning indicators to support the evaluation of the streets’ cleanliness level and to monitor the sweeping operations (Alfarrarjeh et al., 2018; Association des Villes pour la Propreté Urbaine [AVPU], 2016; Lloyd et al., 2018; Lopez et al., 2017; Sevillano and Arévalo, 2017; Zamorano, 2010; Zhang et al., 2019). The existing studies are generally promoted or conducted by municipalities and associations.

In Granada (Spain), the Spanish Federation of Municipalities and Provinces defined an index to evaluate the quality of the street cleaning operations (Zamorano, 2010). The developed cleanliness index varies according to the amount of waste found on the street and depends on the type of pavement, climate conditions and traffic (pedestrian and vehicles), among other factors. The cleanliness index was used to evaluate the dirtiness over 8 days on one street in the city centre of Granada at different times of the day, before and after the sweeping operations. Random areas were considered to obtain an average value of the cleanliness index. An index scale between 70 and 200 was created within this methodology. The study showed cleanliness levels usually higher than 100 before the cleaning operations for the morning and night periods (average index values of 151 and 116, respectively), which classifies the street with ‘low’ and ‘very low’ levels of cleanliness, considering the classification scale established. After the cleaning operations, the cleanliness levels were always ‘very high’, with index values lower than 70 (average index values of 19 and 12 for morning and night periods, respectively). In the afternoon periods, the index values proved to be lower than the rest of the day, both before and after cleaning operations (average index values of 58 and 9, respectively; Zamorano, 2010).

To overcome the constraints that manual methodologies have (time-consuming, prone to errors, absence of real time data), a study performed in China was devoted to develop a cleanliness assessment approach using mobile edge computing and deep learning (Zhang et al., 2019). The study installed high-resolution cameras on vehicles to collect street images, which were further processed to determine the amount and type of waste using the faster region-convolutional neural network. The system developed to identify each waste category could reach an average of 82% of detection accuracy (Zhang et al., 2019). After the waste recognition, the waste units are obtained, and the formula from the previously mentioned study (Zamorano, 2010) can be used to determine the streets’ cleanliness index. However, the recognition system does not include all the types of waste categories, but only the most common ones, which leads to an incorrect value of the street cleanliness level. Besides that, the methodology was always applied on sunny days and thus does not account for other climate conditions (e.g. rainy and cloudy days) that might impact cleanliness levels (Zhang et al., 2019).

In Madrid (Spain), street cleanliness indicators have been used since 2006 to ensure that sweeping street operations are used efficiently and, consequently, an adequate street cleanliness level is provided (Sevillano and Arévalo, 2017). Madrid sweeping companies measure cleanliness indicators monthly to characterise the streets’ dirtiness level by randomly choosing streets and characterising 250 m^2^ samples. Waste pieces are counted considering their size and origin (organic or inorganic; AM, 2013). Around 150 samples are taken monthly between 7 and 22 hours (AM, 2012). In this methodology, a scale for the cleanliness level is not specified; however, a procedure to evaluate the dirtiness level is provided considering the established formula. An acceptable cleanliness level corresponds to a dirtiness level which cannot be higher than 10, on average, for each 250 m^2^ sample and where only 4% of the samples can have a value higher than 20. Unacceptable cleanliness is obtained for values between 10 and 18 for each 250 m^2^ sample, and when the number of samples with a value higher than 20 represents between 4% and 6%. Finally, the cleanliness level is considered critical if the value obtained is higher than 18 for each 250 m^2^ sample and if more than 6% of the samples have a value higher than 20 (AM, 2008).

In Versailles (France), a methodology was also developed to evaluate the streets’ cleanliness level, further used by the cities that joined the AVPU (2016). This methodology has been evolving based on the feedback from its application in different cities. It considers a quantitative assessment of an area by unit-counting of the waste pieces found on the streets. The number of quantified units is normalised to a 100 m^2^ area, and an average dirtiness indicator is further obtained (AVPU, 2016). In 2016, this methodology was updated by including some weighting factors for different waste categories, considering results from the perception of around 200 inhabitants. The methodology is applied by sector of activity (commercial, residential, etc.) whenever possible at the maximum point of dirtiness, considering two-thirds of streets by sector every month (AVPU, 2016). In this methodology, a scale for the cleanliness level is not specified, as the procedure to calculate the cleanliness index considers summing all the normalised units of waste found on each analysed sample (AVPU, 2016). For example, the average values obtained for the animal droppings category and cigarette butts were 0.19 and 0.21, respectively (AVPU, 2016); average values for the different categories are available, allowing other methodology users to evaluate their cleanliness level by comparison.

Considering the relevance of this topic, it is essential to conduct real case studies in different cities known to have variable and unpredictable factors influencing street cleanliness. Considering the existing studies and practical cases, the present study (the first of its kind in Portugal as far as it is known), supported by mathematical modelling, aims to develop and implement a methodology to evaluate the streets’ cleanliness level at Porto city considering a practical quantitative assessment, the embedment of population perception criteria and the establishment of a final scale, easy to interpret.

## Materials and methods

To develop an effective urban cleaning strategy, it is crucial to understand waste distribution patterns, categorise waste types and quantify their presence in different urban settings, including street areas. This study focuses thus on designing a robust methodology to assess street cleanliness, using Porto Municipality as a case study.

One of the fundamental aspects of the methodology developed is the classification of waste categories based on public concerns and existing methodologies. By considering research from waste management entities, and international best practices, a number of waste categories were established. These categories were further classified based on size, allowing for a more detailed and accurate assessment of waste accumulation on city streets. Another essential component is the characterisation of urban zones, as waste generation and accumulation vary depending on the dominant activities in a given area. To address this, the study classified Porto’s streets into a number of distinct areas, considering defined factors. The detail is high to ensure replicability to other urban contexts. The development of the methodology considering the described aspects is detailed as follows.

### Development of the methodology

#### Waste categories

The proposed methodology is based on a field quantification at the city streets and considers selected waste categories and the respective size classification. It considers the main categories found in the streets that are of concern to the general public, supported by the field research developed by *Porto Ambiente* (the company responsible for waste management and urban cleaning at Porto city) and the methodology developed by AVPU (2016). The size distribution for some waste categories is based on the methodology developed by Madrid’s Municipality (AM, 2017). A complete and robust classification was created to characterise all the types of waste found on the streets in a correct and detailed manner. Supplemental Table SM1 resumes the 11 categories defined and the size distribution classification.

#### Zone characterisation

There are several factors which can significantly influence street cleaning. One of the most relevant is the street typology, since it is expected that the nature and the amount of waste varies according to each type. Thus, in order to minimise bias concerning different street types, the work started by defining clearly different street types. This allowed to classify the city and also to select streets for sampling, more representative and thus making the results more accurate. In fact, if commercial activities (e.g. restaurants, supermarkets, etc.) prevail over housing on some streets, the amount and type of waste can significantly differ. Therefore, to conduct an adequate characterisation of the cleaning status of a determined city, it is necessary to: (1) define main areas of activity in which each street is included; and (2) determine their weight in the city. The results of the city might consequently be obtained by using the mean results from the streets characterisation at each area of activity and the respective weight of the activity in the overall city. In this study, nine areas of activity were considered to classify streets, considering *Porto Ambiente’s* know-how. To reduce subjectivity, the street classification must consider the following parameters:

1. Number and type of commercial sites per 100 m length of the street.
2. Number and type of school sites per 100 m length of the street.
3. Number and type of households per 100 m length of the street (considering single-family buildings, multi-family buildings and social housing).
4. Presence of a riverside area.
5. Presence of a touristic area.

To obtain the parameters that allow the allocation of a particular street to one of the nine areas, typical streets of each activity were surveyed (approximately 5 streets per area, on a total of 52). When choosing the streets per area for sampling, a care was taken to consider other important variables such as street construction and topography so that they could properly represent the area and the city. Then, the collected data were used to develop threshold values, which could after be applied to classify all streets into one of the nine defined areas, according to Supplemental Figure SM1.

#### Determination of the activities weight in the city

To determine the percentage of each of the nine defined areas in the total city area (first, using the survey experience and Google Maps), all of the 2,170 streets of Porto city were classified as one of the nine areas. After that, the percentage for each area was obtained, considering the linear length of the streets (based on the municipality’s cartography), as shown in Supplemental Table SM2. Supplemental Figure SM2 shows the city map divided by the respective areas.

### Waste quantification

Each chosen street was divided into samples of 250 m^2^ for non-pedestrian streets and 500 m^2^ for pedestrian streets, taking into account reference values (Lloyd et al., 2018; Lopez et al., 2017). Each sample considered the length and width of the sidewalks, being quantified at least two times on different days of consecutive weeks to improve the quality of the results. All the quantifications were made at the maximum level of dirtiness of the street, as proposed by AVPU (2016). For that, the streets were analysed approximately 1 hour before the cleaning services started the operation, in agreement with the schedule provided by *Porto Ambiente*.

Full sample analysis was divided into five parts. First, the cleaning status of the sidewalks (SW), gardens (GR) and tree grates/flowerpots (TF) were evaluated according to the 11 waste categories and their number, in agreement with the established classification presented in Supplemental Table SM1. The quantification also considered the number of gullies (GU) and litter bins (LT) and their condition. Gullies were classified as ‘With waste’ or ‘Without Waste’ and litter bins as ‘Empty’ (<50% capacity) or ‘Full’ (⩾50% capacity).

During the fieldwork, a one-unit-counting method was used to quantify the waste found. The counting methodology was done using a mobile application (in an Android device), created exclusively for this purpose. The mobile application served only to support an easy data gathering/collection; thus, its development and the intellectual property is outside the scope of the present study. The application was developed using the Android Studio, and collected data, considering the counted units per type of waste, was stored in a CSV file, being subsequently exported for data treatment in terms of equivalent units.

For that, after defining weighting factors for each category/size of waste, equivalent units for the different types of waste could be obtained (Supplemental Table SM3). These weighting factors were defined considering the fieldwork experience and population surveys carried out in a previous study (Pereira, 2018).

A total of 52 streets (783 samples; 116,780 m^2^) were monitored (listed in Supplemental Table SM4), which are distributed by area of activity as follows: 10 for commercial/touristic (CT) area, 5 for commercial (C) area, 7 for residential 1 (R1) area, 6 for residential 2 (R2) area, 8 for residential 3 (R3) area, 5 for commercial/residential (CR) area, 4 for school (S) area, 5 for school/residential (SR) area and 2 for riverside (Riv) area (Supplemental Figure SM3). The field quantification lasted 3 months, being conducted during weekdays between the 1st of May and the 31st of July 2019. Although not very recent, the general maintenance of the city metabolism and its characteristics (in structures, population distribution and city areas) indicate that the cleaning variables studied; thus the results obtained should not be affected presently. In Porto Municipality, a slight increase in population has been observed (e.g. 2.4% from 2022 to 2023), although a decrease is projected up to 2060 according to Porto Economic bulletin 2023. The main urban developments in last years according to the municipal plans relate to land use, housing, urban regeneration and innovation. In this period, there were some green infrastructure development (Parque da Asprela), initiatives of social integration and rehabilitation (e.g. rehabilitation of the Bolhão Market). In addition, metro expansion works have been underway, and smart solutions have been implemented (e.g. for energy in buildings and for mobility). This is aligned with previous developments for the region; thus, significant changes are not expected to the present reality. Last changes to Porto master plan date from 2025 (warning no. 14529/2025/2). Relevant additional parameters concerning methodology to minimise biases some relevant factors are described as follows. To minimise climatic bias, sampling was conducted during stable weather conditions, avoiding days of rain and strong winds. Surveys were conducted whenever possible during time periods where a higher amount of people could be found in the streets to ensure the highest response rate possible; in addition, to ensure proper correlation with the sampling data, the population surveyed was that passing in the area which was being quantified. The quantification was done before the timing of the cleaning services, to ensure similar conditions which represented the highest dirtiness level.

### Cleaning indicators

To obtain the overall cleanliness index, it was necessary to establish specific parameters and indicators. A resume of the purpose of each one is described as follows: (1) waste equivalent units (*R_i_*) – convert the results of different areas to a common base; (2) cleanliness indicator for each waste category (CL_*i*_) – measures the cleanliness associated to each waste category on a percentage scale; (3) waste category annoyance indicator (*w_i_*) – incorporates public perception regarding the nuisance of each waste category; (4) cleanliness indicator for disposal places (CL_*x*_) – considers the cleanliness level of specific waste disposal places; (5) waste disposal place annoyance indicator (*P_x_*) – incorporates public annoyance specifically related to the different disposal places.

The overall cleanliness level indicator (CL) aggregates all parameters and indicators and provides a single, comprehensive cleanliness percentage for a given area or city.

It is essential to highlight that these indicators were developed taking Porto city as an example.

The proposed methodology for each parameter and indicator is described in more detail in the following subsections.

#### Waste equivalent unit (*R_i_*)

The quantification results for the analysed samples of 250 or 500 m^2^ should be firstly standardised to 100 m^2^ and consider the smaller size of waste so that all results can be comparable. After this procedure, the waste equivalent unit (*R_i_*) can be obtained using equation (1) and Supplemental Table SM3.

(1) $$R_{i}=\frac{\sum_{j=1}^{3}\left(p_{ij}\times r_{ij}\right)}{A}\times 100$$

where *A* is the area (m^2^) of the sample, *p* is the size factor (Supplemental Table SM3), *r* is the units of waste found, *j* is the waste size (1 for small, 2 for medium and 3 for large) and *i* is the waste category.

#### Cleanliness indicator for each waste category (CL_*i*_)

This indicator is measured in percentage (%), considering the minimum and the maximum amount of waste of each category (in equivalent units) which can be acceptable in a 100 m^2^ area. In addition to the amount/typology of waste found, it is also essential to know the value of CL_*i*_ for each waste category individually to realise if some category compromises the street cleanliness level. Based on a previous study, minimum and maximum values were obtained to establish a linearity range based on the sampling results (Pereira, 2018). Supplemental Table SM5 shows the limits and respective linear adjustments obtained after field studies. If the *R_i_* value is less than the minimum threshold, the CL_*i*_ indicator is 100% (a very clean area for that waste category). Otherwise, if the *R_i_* value exceeds the maximum limit, the CL_*i*_ indicator is 0% (very dirty area). For *R_i_* values between the two defined limits for each waste category, a linear adjustment was used to calculate the CL_*i*_ indicator.

#### Waste category annoyance indicator (*w_i_*)

A waste annoyance indicator for each category was obtained from a population survey, detailed in (Pereira, 2018) and section ‘Population survey’. The population survey was performed to understand which waste categories are a problem to the users in terms of street cleanliness. The answers obtained (classified from 1 to 5) were summed, and a weighted average was determined for each waste category for each disposal place (SW, TF and GR). To determine the cleaning indicator, the *w_i_* parameters were normalised, being their sum equal to 1. Supplemental Table SM6 shows *w_i_* values obtained for sidewalks, tree grates/flowerpots and gardens.

#### Cleanliness indicator for disposal place (CL_*x*_)

The cleanliness indicator for each disposal place is measured in percentage (%). It can be helpful to understand whether samples or sample sets have a problem with a specific place where waste is disposed. As mentioned before, these places can be sidewalks, gardens, tree grates/flowerpots, gullies or litter bins. To obtain the indicator value for sidewalks (CL_SW_), gardens (CL_GR_) and tree grates/flowerpots (CL_TF_), equation (2) was applied.

(2) $$CL_{x}=\Pi_{i=1}^{11}({\mathrm{CL}}_{i}^{w_{i}})$$

where CL_*i*_ is the indicator value for each waste category *i*, *w_i_* is the annoyance index for that category and *x* is the disposal place. This indicator is calculated using the product operator instead of the sum. In this way, for 0% values of CL_*i*_, for one or more waste categories, the result of CL_*x*_ will always be 0% too. Thus, if some waste category exceeds the maximum limit settled, the cleanliness level for that category is unacceptable (zero), as well as the cleanliness of the place of its disposal.

For the gullies’ indicator, equation (3) was applied. This indicator is the ratio between the number of clean gullies and the total number of gullies analysed in that sample. Before the calculation, these numbers were standardised to the number of gullies (clean and total) per 100 m^2^.

(3) $${\mathrm{CL}}_{\mathrm{GU}}=\frac{N_{\mathrm{GU}\;\mathrm{clean}}}{N_{\mathrm{GU}\;\mathrm{total}}}\times 100$$

For the litter bins’ indicator, equation (4) was applied. This indicator is the ratio between the number of empty litter bins (<50% capacity) and the total number of litter bins analysed in that sample. Before the calculation, these numbers were also standardised to the number of litter bins (empty and total) per 100 m^2^.

(4) $${\mathrm{CL}}_{\mathrm{LT}}=\frac{N_{\mathrm{LT}\;\mathrm{empty}}}{N_{\mathrm{LT}\;\mathrm{total}}}\times 100$$

#### Waste disposal place annoyance indicator (*P_x_*)

Waste disposal place annoyance indicator was obtained from the inquiry answers for question 2, more detailed in section ‘Population survey’ and described in a previous study (Pereira, 2018). This question was performed to understand which place is, for the population, a problem in terms of street cleanliness. All the answers obtained (classified from 1 to 4) were summed, and a weighted average was done for each disposal place. As for the *w_i_* indicators, *P_x_* indicators must also be converted in a 0 to 1 scale, where the sum of the five-place indexes must be 1. As mentioned before, gullies were not included in the survey; in such case, the attributed value was the lowest one (obtained for the tree grates/flowerpots). Supplemental Table SM7 shows the *P_x_* values obtained.

#### Cleanliness level indicator (CL)

The cleanliness indicator is measured in percentage (%), and it is the one to use to fully evaluate the cleanliness level (CL) of some sample, street, area, city, etc. This is determined using all the other developed indicators, as shown in equation (5).

(5) $$\mathrm{CL}=\frac{\sum \left({\mathrm{CL}}_{x}\times P_{x}\right)}{\sum P_{x}}$$

This indicator is calculated using a sum instead of a product operator. In this way, if one or more values of CL_*x*_ are 0%, the CL level may not be zero because it will depend on the other existing factors.

A comprehensive summary table of all indicators with definitions, formulas and units is presented in Supplemental Table SM8.

### Population survey

The perception of the population about the street cleaning status was evaluated through an inquiry of three simple questions performed during field quantification at the selected streets. The first question was related to the degree of annoyance of the different waste categories. In this question, 12 different photos were shown to the surveyed people, one of each category (except for Incrustations, where two were included). People should classify each waste category on a scale of 1–5, where 1 was ‘It does not bother me at all’ and 5 ‘It bothers me a lot’. The second question was related to the degree of the bother of the dirty place in the street. In this question, four different photos were shown, one of each place where waste can be disposed (sidewalk, garden, tree grate and litter bin). People should order each disposal place from the one that bothers less (1) to the one that bothers more (4). The third question was related to the population perception of the overall cleaning degree of the street. It was asked to surveyed people to look at the street at that precise moment and classify its cleanliness degree on a scale from 1 to 5, where 1 is ‘Very dirty’, and 5 is ‘Very clean’.

These questions were randomly made to the overall population, including at the streets, at the company, at the faculty and on social occasions. When doing the questionnaire, there was a care to consider different ages although the focus was in accessing people passing in the streets under assessment. Data concerning age was not considered. The gender data were collected. The results of the surveys relate to two phases. The first phase, resulted in the annoyance indices (question 1 and 2), and specific results are presented in Pereira (2018). Results from question 3 in the first phase were considered of low quality; for this reason, an additional survey phase was carried out to increase the accuracy of the model, focusing only on question 3, results of which are presented in this study. Overall, about 3,000 people were enquired, including city residents and tourists, from all age groups and genders, as equitably as possible. A correspondence table was created based on the relation between survey answers and CL values obtained from the sampling process.

### Parameters optimisation

The proposed methodology is based on a field quantification at the city streets and considers selected waste categories and the respective size classification as well as disposal place. It also considers specific annoyance factors gathered from surveys both for waste categories and the disposal place. After applying the developed methodology, it is possible to obtain a cleanliness level indicator. However, considering that the objective is to improve cleanliness services in agreement with population perception and well-being, an additional optimisation of parameters was made to the annoyance factors so that the final result of the CL values were as close to people’s survey answers possible. In fact, although each individual person might consider a certain waste category or place causing a high annoyance, it might occur that it does not mean that the citizen considers that a street containing for example certain waste is not clean. To do so, the surveys included not only assessment of each category and place annoyance but also the classification of the degree of cleanliness of the streets.

Therefore, the parameters *P_x_* and *w_i_* were optimised. In the algorithm, each parameter was optimised individually, following an iterative process. A range for each parameter value was defined (depending on the parameter value), and several values were tested within this range. The value corresponding to the minimum deviation between the methodology’s and the survey’s values was then selected (objective function was defined as the minimisation of the sum of the squared deviations). This process was repeated for all the parameters and ended when the objective function had a relative variation (between iterations) lower than 0.1%.

## Results and discussion

### Waste quantification

**Figure 1.**
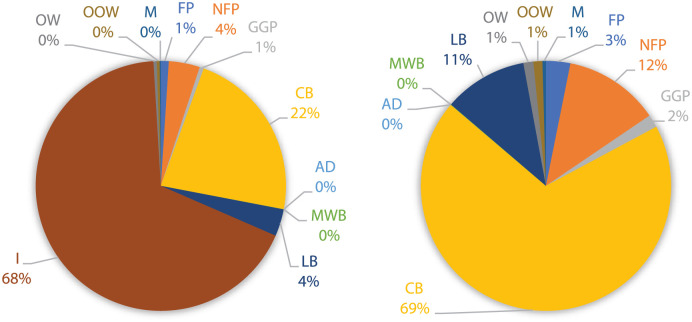
Waste distribution on sidewalks for commercial/touristic area (left); and, without incrustations (right). FP: food packaging; NFP: non-food packaging; GGP: glass and glass pieces; CB: cigarette butts; AD: animal droppings; MW: mixed-waste bags; LB: leaves and branches; I: incrustations; OW: organic waste; OOW: other type of organic waste; M: metals; SW: sidewalks; TF: tree grates/flowerpots; GR: gardens.

**Figure 2.**
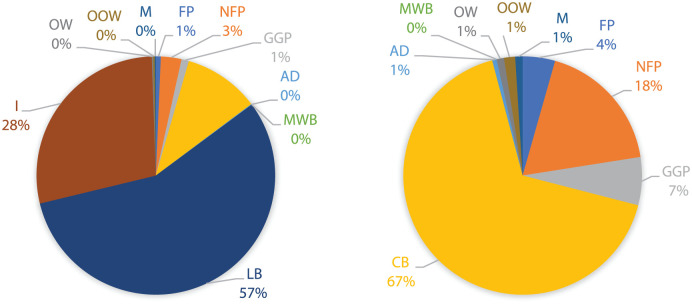
Waste distribution on sidewalks for residential 1 area (left); and, without incrustations and leaves and branches (right). FP: food packaging; NFP: non-food packaging; GGP: glass and glass pieces; CB: cigarette butts; AD: animal droppings; MW: mixed-waste bags; LB: leaves and branches; I: incrustations; OW: organic waste; OOW: other type of organic waste; M: metals; SW: sidewalks; TF: tree grates/flowerpots; GR: gardens.

After the fieldwork and the sampling process, the percentual weighting values were obtained for each waste category in nine city areas. On sidewalks, per-unit waste, the highest incidence categories for most city areas were incrustations (I) and cigarette butts (CB). In commercial areas, these categories were found more frequently (68% and 22%, respectively) than in residential ones (28% and 10%, respectively), representing more than double. CB are a sweeping urban problem in Porto city, representing, on average, 20–30% of the total amount of waste found per 100 m^2^ street. In residential and school areas, leaves and branches (LB) category percentages are between 25% and 50%, representing three times more than in commercial areas (with percentages that do not exceed 15%). This category represents a problem for sweeping services, mainly during the leaf-falling period, as they may clog the gullies compromising the run-off water to flow through them. In a transversal way, the following category with more expression is non-food packing (NFP). On the other hand, the least common categories for all the city areas are metals (M), other types of organic waste and mixed-waste bags. The results obtained for the CT and R1 areas are shown in Figures 1 and 2.

### Cleanliness indicator for each waste category (CL_*i*_)

The CL_*i*_ values were obtained for the three disposal places analysed (SW, TF and GR) and for each city area. Supplemental Table SM9 shows the respective graphics, as well as the relative distribution. In a transversal way, CL_*i*_ values for animal droppings (AD) and NFP categories are the ones more affected, especially for tree grates and gardens. As expected, this happens more frequently in residential than in commercial areas.

For residential areas, CL_*i*_ value decreases with the increase of people influx. Thus, R1 areas are cleaner than R2 areas, which are cleaner than CR areas, generally for all the categories (R3 areas were excluded from this reasoning because they are atypical situations – social-housing). For Porto city, as for most areas individually, AD and NFP are the categories that represent cleaning problems. Even though not so evidently, cigarette butts and incrustations are also a cleaning problem at SW.

### Cleanliness indicator for each disposal place (CL_*x*_)

Table 1 shows the values for the cleanliness level of the five disposal places analysed during the fieldwork for each city area and the total obtained for Porto city. Supplemental Table SM10 shows the respective graphics, as well as the relative distribution. For most areas, and whatever the relative frequency, in terms of cleanliness levels, the most problematic places are, successively, GR, GU and SW. On the other hand, LT is the least problematic one. Concerning SW, cleanliness levels for commercial areas were higher than the achieved values for residential areas. R2 is the most concerning area, mainly due to the results obtained for LB at SW and AD at TF and GR. By contrast, Riv area is the least problematic area. The applicability of the methodology as a diagnostics tool should not be neglected; thus, it is identified the need to intervene in certain areas. As relevant suggestions to problematic areas such as residential and school zones, specific awareness campaigns are advised, designed for the target population a specific waste types, namely cigarette butts and animal droppings.

**Table 1.** Cleanliness levels (CL_*x*_, %) results, before cleaning operations, for the city areas and disposal areas as well as for Porto city.

City Area	CL_SW_	CL_TF_	CL_GR_	CL_GU_	CL_LT_
CT	85.11	84.90	98.10	97.15	71.73
C	79.04	84.60	65.13	61.91	77.22
R1	28.47	96.03	62.20	53.75	100.00
R2	22.14	7.51	17.17	17.50	35.68
R3	26.69	87.75	31.07	64.61	100.00
CR	72.69	39.25	82.64	55.38	80.91
S	58.74	82.61	34.76	40.22	60.86
SR	48.91	32.36	21.86	62.36	73.12
Riv	89.46	96.73	93.21	81.32	81.58
Porto city	43.39	64.07	53.51	51.82	78.34

CT: commercial/touristic area; C: commercial area; R1: residential 1 area; R2: residential 2 area; R3: residential 3 area; CR: commercial/residential area; S: school area; SR: school/residential area; Riv: riverside area; SW: sidewalks; TF: tree grates/flowerpots; GR: gardens; GU: gullies; LT: litter bins.

### Cleanliness indicator (CL)

Table 2 presents the key results obtained for CL values at Porto city and its nine areas. CL value for the whole city was obtained considering the weight of each area in the city (Supplemental Table SM2) and the maximum dirtiness level. As seen in Table 2, CT and Riv are the cleanest city areas, with CL values of 85% and 86%, respectively. On the other hand, R3 (social housing) and R2 (multi-family buildings) are the less clean areas, with CL values of 47% and 54%, respectively. These low values prove that social-housing areas, as well as areas with a lot of resident people influx, are in more need of actuation to the sweeping cleaning services. However, in the CT area, despite its large population influx, CL value is one of the highest, which means these streets show a high cleanliness level. Thus, its higher sweeping frequencies appear to be well-adjusted to the needs. In most cases, higher sweeping frequencies result in higher CL values.

**Table 2.** Cleanliness level (CL%, before cleaning operations) and standard deviation (*s*, %) results for Porto city and respective areas.

City Area	CL (%)	*s* (%)
Commercial/touristic area	85	15
Commercial area	76	17
Residential 1 area	60	15
Residential 2 area	54	18
Residential 3 area	47	14
Commercial/residential area	68	19
School area	58	20
School/residential area	55	23
Riverside area	86	13
Porto city	61	17

In residential and school areas, a large amount of animal droppings were found mainly due to the high frequency of gardens and tree grates. As this category has the highest annoyance level to the population (Pereira, 2018), it largely affects these streets’ CL values.

For Porto city, CL value was 61%, considering each area weight (Supplemental Table SM2). Considering all the areas equitable, CL value would be 74%, much higher than the one obtained. This occurs due to the low CL values for the main city areas (residential areas). However, the results are not unsatisfactory since they result from measures at the highest dirtiness level.

### Population survey

**Figure 3.**
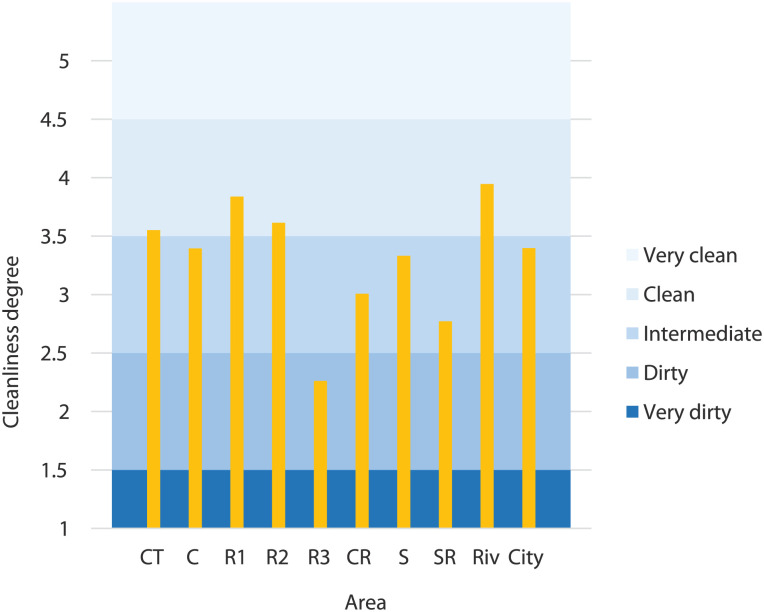
Weighting average of the survey answers to question 3, for Porto city and each city area. CT: commercial/touristic area; C: commercial area; R1: residential 1 area; R2: residential 2 area; R3: residential 3 area; CR: commercial/residential area; S: school area; SR: school/residential area; Riv: riverside area.

Of the surveyed 3,269 people, 54% were women, and 46% were men. The answers distribution is shown in Supplemental Figure SM4. Generally, the population considers the streets at intermediate and clean levels (67% of the answers). The very dirty level only represents 5% of the answers of the inquired population. With these answers, weighting averages were obtained for Porto city and each of the nine areas, being presented in Figure 3.

In people’s opinion, the cleanest areas are Riv and R1 areas, with 3.98 and 3.84 (on a scale from 1 to 5), respectively. The less clean areas were R3 and SR, resulting in 2.26 and 2.77 cleanliness degrees, respectively. The results for Riv and R3 agree with the values reported in section ‘Cleanliness indicator (CL)’, since the higher CL values were obtained for the Riv area and the lower CL values for R3 area. Therefore, Porto city has a 3.37 out of 5 cleanliness degree for the inquired people, classified as intermediate in terms of cleanliness degree (details further).

After the surveys, a table with the cleanliness degree values allocated to CL values was created, according to the results obtained, presented in Table 3. This allocation was based on the relationship between survey answers and CL values obtained from the sampling process. For example, for the intermediate level (from 2.5 to 3.5), most CL values obtained for the analysed streets were between 50% and 70%, which explains the allocated array, as seen in Table 3. The same occurs for the other arrays. However, as values between 1 and 1.5 were not obtained from the survey, a 30% value was defined for this range because this is, on average, the smaller CL value obtained for the analysed streets. A similar procedure was adopted for the 4.5 to 5 range.

**Table 3.** Correspondence between CL values and the survey answers.

Cleanliness degree ranges	CL
Very dirty (1–1.5)	0 ⩽ CL < 30
Dirty (1.5–2.5)	30 ⩽ CL < 50
Intermediate (2.5–3.5)	50 ⩽ CL < 70
Clean (3.5–4.5)	70 ⩽ CL < 90
Very clean (4.5–5)	90 ⩽ CL ⩽ 100

CL: cleanliness level.

**Figure 4.**
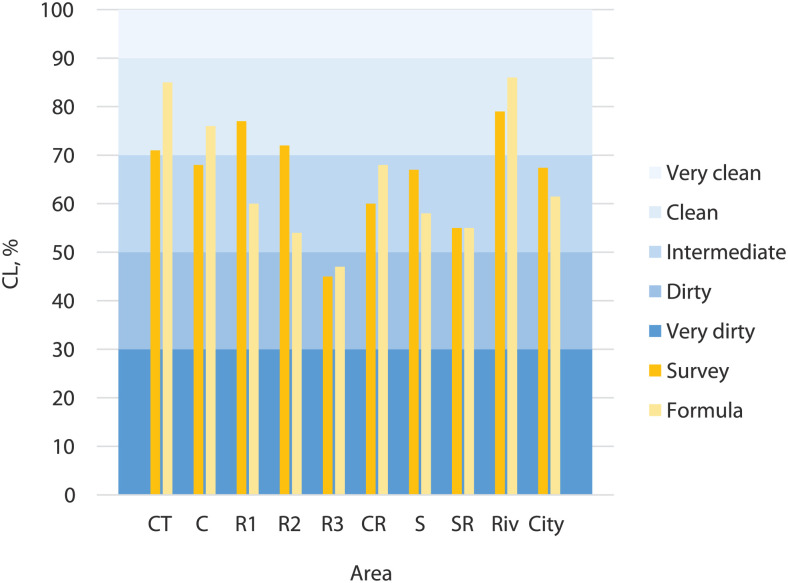
Relation between survey answers and CL values. CL: cleanliness level; CT: commercial/touristic area; C: commercial area; R1: residential 1 area; R2: residential 2 area; R3: residential 3 area; CR: commercial/residential area; S: school area; SR: school/residential area; Riv: riverside area.

This correspondence table allows the transformation of survey answers into their respective CL values. These values are shown in Figure 4 for each city area. In this way, people’s opinion on the streets’ cleanliness degree and its actual cleanliness level obtained from the created methodology can be compared.

People’s opinions are generally more stringent than the cleanliness levels obtained from the methodology employed, with exceptions for residential areas 1 and 2, and for the school area. In these cases, surveyed people classified the streets more positively; therefore, CL values obtained are so not well adjusted. In most cases, the differences found are less than 10% and globally represent only 6%. Thus, and considering the type of variables under study and the high complexity associated, the discrepancies are not considered high. However, the authors consider that a robust methodology should privilege populations opinion. Thus, a subsequent development of the methodology included a parameter optimisation phase to adjust the model’s values according to people’s perception ‘that better reflect the streets’ cleanliness degree (see section ‘Parameters optimisation’). Following subsection describes the obtained results.

### Parameters optimisation

**Figure 5.**
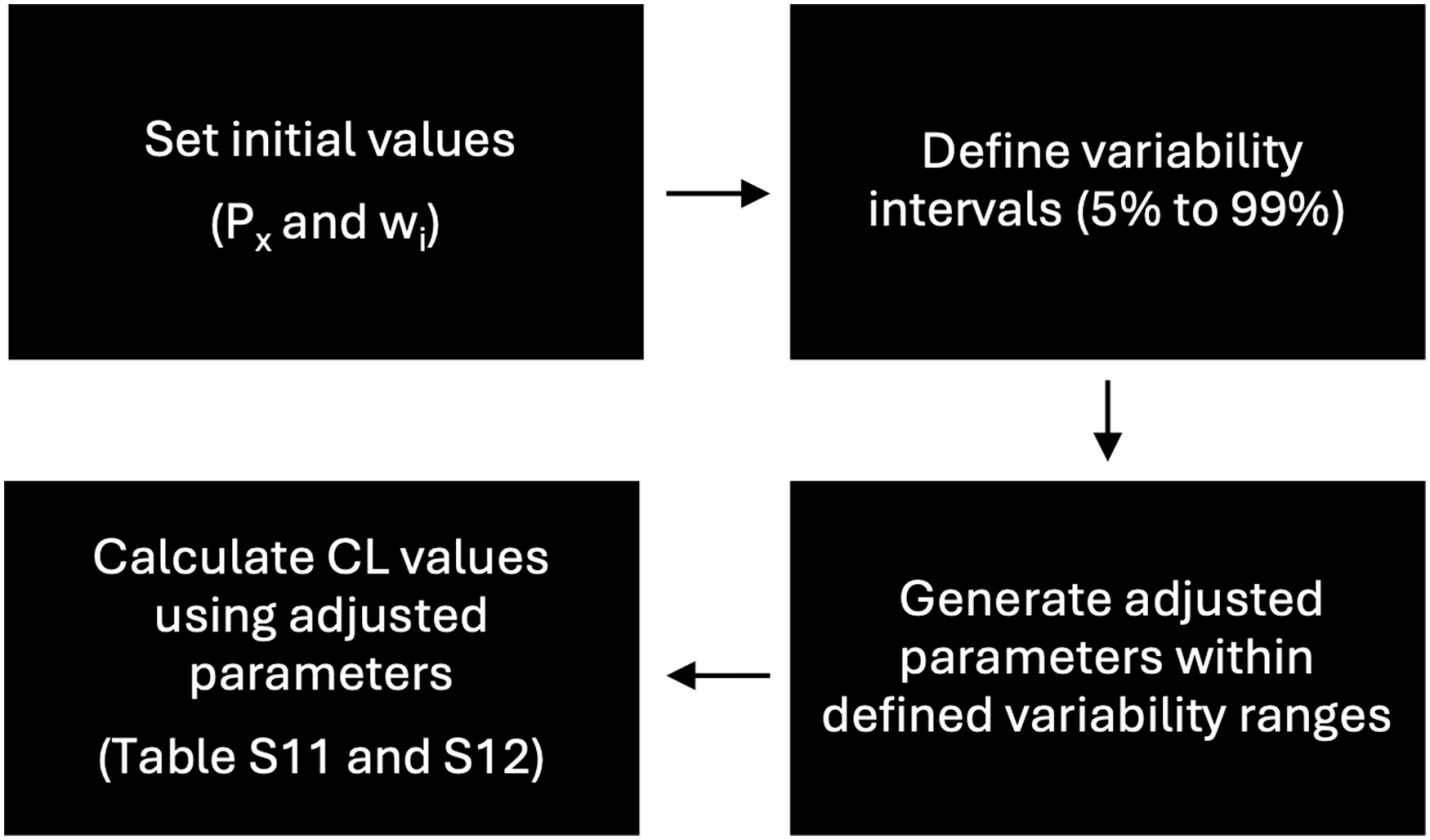
Parameters optimisation flow diagram.

Figure 5 shows the parameters optimisation flow diagram.

Supplemental Tables SM11 and SM12 show the original parameters used for CL calculations and the ones obtained from the adjustments. These were made for *P_x_* and for *w_i_* values, with optimisation intervals corresponding to 5% and 99% of the parameters’ variability. Adjustments made for more than 40% of parameters’ variability do not suit these methodology requirements because GU weighting factors become higher than SW ones. This cannot happen because, generally, the cleanliness of sidewalks is more important than gullies’ cleanliness, so the weighting factors cannot be depreciated. For *w_i_* values, parameters’ variabilities higher than 20% do not suit these methodology requirements. This happens because, for some waste categories, as NFP and CB, *w_i_* values are too depreciated. This cannot happen, especially for these two categories because these represent important cleaning problems, as seen in section ‘Waste quantification’. The AD category (the most important in people’s opinion) is also depreciated for higher variability percentages. Thus, the variability of the parameters was considered acceptable until a 10% variability, inclusively.

The differences found between the original and the adjusted parameters were not significant for the acceptable optimisation range, so CL values do not improve significantly, as shown in Table 4. Although specific statistic results cannot be detailed, since the parameter optimisation was done using optimisation excel/embedded tools, using defined requisites (range in agreement with the parameter characteristics and minimisation of the sum of the squared deviations), the low errors associated are clear. In fact, for most city areas and the city itself, CL value differences of 1% and 2% were obtained for 10% and 20% parameter variability, respectively. These differences are more pronounced for residential areas, mainly residential 1 area, with a 5% CL difference. This is relevant because these areas were where survey answers presented higher differences from CL values. The found similarities support the use of the developed methodology to obtain the streets’ cleanliness level because CL values generally match people’s opinions. In addition, results highlight the complexity and the relevance of field validation, since optimisation was impaired due to unacceptability from practical knowledge (e.g. ‘the cleanliness of sidewalks is more important than gullies’ cleanliness, so the weighting factors cannot be depreciated’).

**Table 4.** Relation between CL values obtained with the original parameters, for 10% and 20% of parameters’ variability.

City Area	Original (%)	10% (%)	20% (%)
Commercial/touristic area	85	85	85
Commercial area	76	76	76
Residential 1 area	60	63	65
Residential 2 area	54	54	55
Residential 3 area	47	48	50
Commercial/residential area	68	67	67
School area	58	59	59
School/residential area	55	55	55
Riverside area	86	85	85
Porto city	**61**	**62**	**63**

CL: cleanliness level.

### Methodologies’ comparison

Spanish and AVPU methodologies’ are well defined in terms of waste characterisation and its counting methods. Through the knowledge acquired from this methodology allied with Porto Ambiente’s know-how, 11 waste categories were defined for this study. Besides the waste characterisation, this study also defined unequivocal factors to distinguish waste pieces by size. In contrast, Granada’s methodology may further specify the distinction between the different sizes of waste and create more detailed parameters. Considering the high complexity associated and the number of defined categories and areas to be evaluated, the authors considered that more detailed parameters would further increase complexity without apparent expected advantages from the application perspective defined (improve waste cleaning services).

Concerning the sampling procedure, this study considers areas of 250 or 500 m^2^ (for non-pedestrian and pedestrian streets, respectively) as occurs in the Spanish methodologies. For the AVPU methodology, areas between 1000 and 3000 m^2^ were considered. However, selecting samples with such high dimensions can lead to a lack of consistency in waste characterisation; thus, the present methodology used smaller areas aiming the highest precision possible. This was done to avoid that a certain sample is classified with a good cleanliness level, even though there are areas included in those larger samples where the waste left on the streets is more concentrated, leading to low representativeness.

During the fieldwork, the Spanish methodologies consider the disposal place where the waste can be found including the criteria to determine cleanliness indexes. The created methodology also considers these factors, and additionally, indicators for each waste category (CL_*i*_) were developed as well as for each disposal place (CL_*x*_), besides the indicator for the street cleanliness level itself (CL). Instead, AVPU’s methodology does not consider the waste disposal place to calculate the cleanliness level. The present methodology aims to support actions of improvement in the services; thus, the place and specific type of waste is highly relevant to promote target measures of improvement.

Regarding the influence that each city area has on waste production, in terms of amount and waste categories, AVPU methodology differs from the Spanish one because the calculation of the cleanliness level considers if some areas correspond to, for example, a commercial or a residential area. During this study, the distinction between city areas was also considered, with the classification of all the Porto city streets, and the cleanliness levels were obtained considering these different areas.

Granada’s methodology considers, in the created cleanliness level formula, different correction factors, such as the pavement state of conservation and climatic factors. These might be important factors that could be included in future work that would improve the quality of the results depending on the city; however, climatic factors influence was minimised in the methodology developed; thus, the sampling methodology should be updated to include this variable. In addition, the different city areas analysed have in general the same city development parameters; thus, pavement state of conservation is not expected to present a high influence in the results obtained. Another relevant aspect relates to the possibility of construction work on street infrastructure which might affect street cleanliness, a factor that was not considered in the present study because it falls outside the scope. It might be relevant to consider its influence in future studies, also in connection with the waste management services.

In sum, the developed methodology is robust, broad and includes weighting factors for each waste category and waste disposal place, based on population and waste management technicians’ perception. Through the application of this methodology, a percentual value of cleanliness is obtained, which is considered to be easy to interpret because it has an associated scale (from 0% to 100%), unlike the Spanish and AVPU methodologies. Besides that, the cleanliness indicator for each disposal place results from a product operator, considering the relevance of each waste category. When the cleanliness level reaches an unacceptable level, the cleanliness value for that disposal place is zero. It should also be mentioned that the final cleanliness level indicator is obtained through a sum operator, which is adequate considering the importance of each cleanliness indicator. The mentioned factors distinguish this methodology from the other referenced methodologies, making it unique and relevant from a scientific and practical perspective.

Considering that the tool has a highly detailed basis, although developed in application to Porto city, the potential adaptability of the methodology to other urban contexts is considered to be high. This occurs since general parameters, street types and city areas and related infrastructures transversally exist in the different urban contexts. However, in order to ensure high level of accuracy and applicability it is suggested that cities develop internal mechanisms of calibration and adaptation of the methodology. This, specially concerning citizens perspectives on cleanliness levels and also cleaning performance parameters defined in each city, which are considered to be the most city-specific parameters. In addition, sampling and accounting pilots should be made to confirm the robustness of the defined methodology in each city. As the study presents a high level of detail in the methodology developed, cities and different urban contexts have the required basis to use and adapt it to their real contexts.

Finally, it should be highlighted that, compared to the other cities mentioned previously, Porto city presents its specific challenges regarding street cleanliness due to its steep topography, narrow historical streets and a highly concentrated urban tourism sector. Moreover, the city experiences high daily inflows of tourists and commuters, which increases pressure on cleaning services and poses a challenge for their planning and management. These characteristics make Porto a relevant and unique case study for assessing street cleanliness.

## Conclusions

Generally, the developed methodology to determine urban cleaning indicators for Porto city is robust and easily shows the people’s perception of the streets’ cleanliness level. The quantification and calculation methods defined within this methodology made the cleanliness levels measurements easier and more automatised. The analysis made to the different city areas allowed to identify areas for improvement of daily cleaning frequency, in terms of waste categories, such as cigarette butts, animal droppings or leaves and branches, and also about sweep frequencies, especially in residential areas.

The methodology is ready to be implemented to monitor and quantify Porto city’s cleanliness degree, and it can help to establish goals and reference values as well as to control the quality of the sweeping operations. Naturally, there is always room to enhance the feasibility of the results and reduce the errors, improving the sampling process progressively and the people surveying, not only about the streets’ cleanliness level, but also about the ‘annoyance index’ of each waste category. However, a significant change in the results obtained is not expected, considering the depth of the current study, and the complexity associated with the city’s dirtiness generation.

Finally, the study highlights future scientific perspectives in terms of areas of development and improvement. Namely, it would be relevant to assess the complementarity between the use of the application and other tools to support the methodology, namely considering other mobile apps, the use of images and videos and the respective processing, incorporation of artificial intelligence and the calibration of the tool to other urban contexts. Finally, the follow-up of trends of cleanliness assessment and population behaviours throughout time would be relevant.

## Glossary of notations

### Abbreviations

AD: animal droppings

AVPU: Association des Villes pour la Propreté Urbaine

C: commercial area

CB: cigarette butts

CL: cleanliness level

CR: commercial/residential area

CT: commercial/touristic area

FP: food packaging

GGP: glass and glass pieces

GR: gardens

GU: gullies

I: incrustations

LB: leaves and branches

LT: litter bins

M: metal

MW: mixed-waste bags

NFP: non-food packaging

OOW: other types of organic waste

OW: organic waste

R1: residential 1 area

R2: residential 2 area

R3: residential 3 area

S: school area

SR: school/residential area

SW: sidewalks

Riv: riverside area

TF: tree grates/flower pots

### Variables

*A*: sample area (in m^2^)

CL: cleanliness level (in %)

CL_*i*_: cleanliness level for each category *i* (in %)

CL_*x*_: cleanliness level for each waste disposal place *x* (in %)

*N*_GU clean_: number of gullies clean

*N*_GU total_: total number of gullies

*N*_LT empty_: number of litter bins empty

*N*_LT total_: total number of litter bins

*p_ij_*: size factor for each waste category *i* and size *j*

*P_x_*: weighting factor for each waste disposal place *x*

*R_i_*: amount of waste by category *i* (number/100 m^2^)

*r_ij_*: units of waste found by category *i* and size *j*

*w_i_*: annoyance index by waste category *i*

### Index

*i*: waste category

*j*: waste size

*x*: waste disposal place (e.g. sidewalks, gardens)

## Supplemental Material

sj-docx-1-wmr-10.1177_0734242X251387004 – Supplemental material for Development of a tool to enhance street cleaning service efficiency: The case study of Porto city, Portugal Supplemental material, sj-docx-1-wmr-10.1177_0734242X251387004 for Development of a tool to enhance street cleaning service efficiency: The case study of Porto city, Portugal by Maria Guedes, José C M Pires, Fernando G Martins, Carolina S Lucas, Hélder Claro, Manuel Fernando R Pereira and Joana M Dias in Waste Management & Research
