# Supplementary material for: Development of a tool to enhance street cleaning service efficiency: The case study of Porto city, Portugal
Source: Waste Manag Res. 2025 Nov 11;44(4):376–88. doi: 10.1177/0734242X251387004 (PMC12988005; doi:10.1177/0734242X251387004)
Supplement: sj-docx-1-wmr-10.1177_0734242X251387004 – Supplemental material for Development of a tool to enhance street cleaning service efficiency: The case study of Porto city, Portugal [file sj-docx-1-wmr-10.1177_0734242X251387004.docx]

**Supplementary Material**


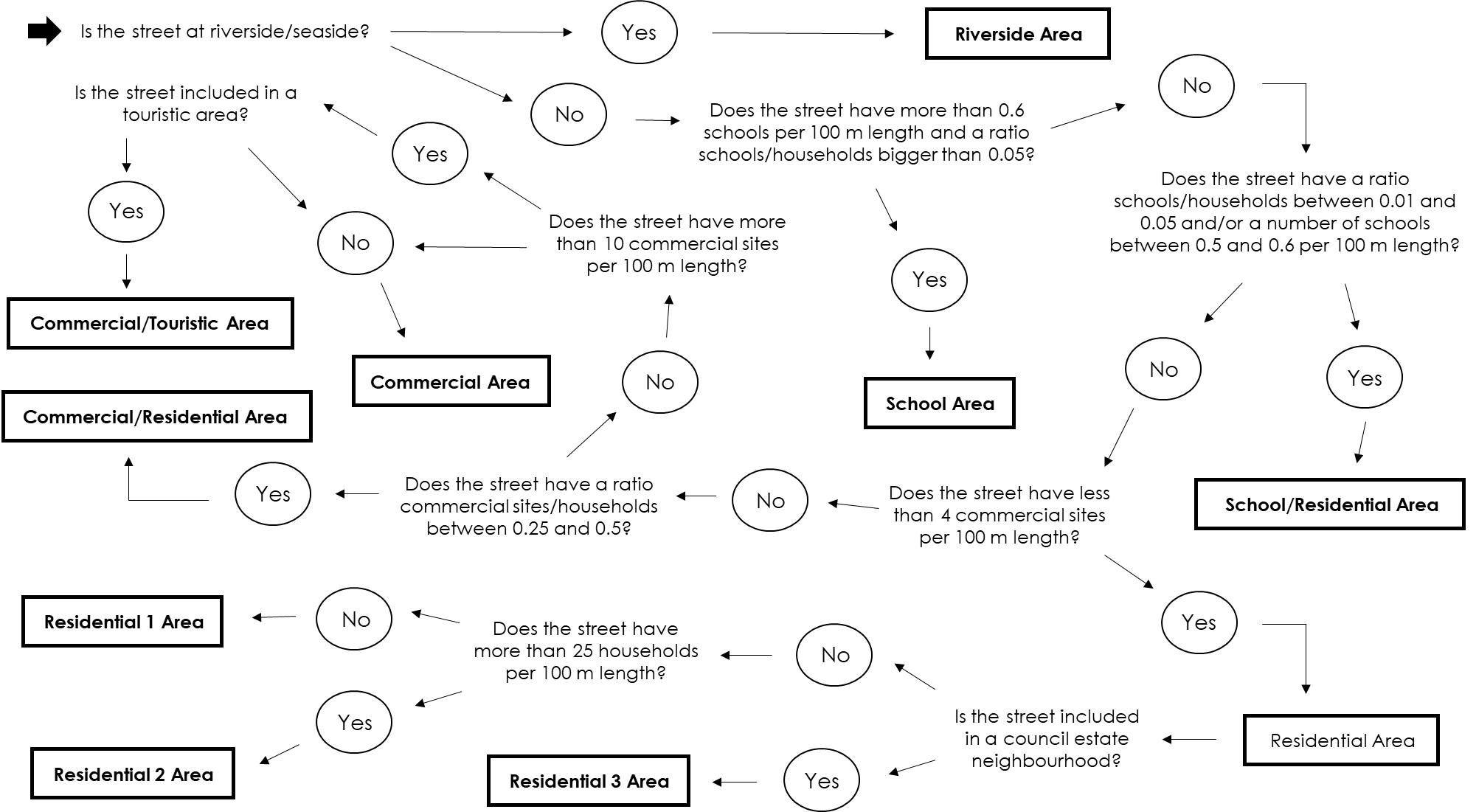


Figure SM1 – Schematic representation of the decision process and parameters used to classify the street typology

Note: The counting process must be done in “equivalent units”, so the values obtained can be comparable. For households, it must be counted 1 unit for every doorbell. For schools, it must be counted 0.5 units for nursery or library, 1 unit for kindergarten, school, or institute, 1.5 units for high school and 2 units for university, college or school residence. For commercial sites, it must be counted 1 unit for small business (ex. Café, office), 3 units for supermarket and 5 units for shopping centre.


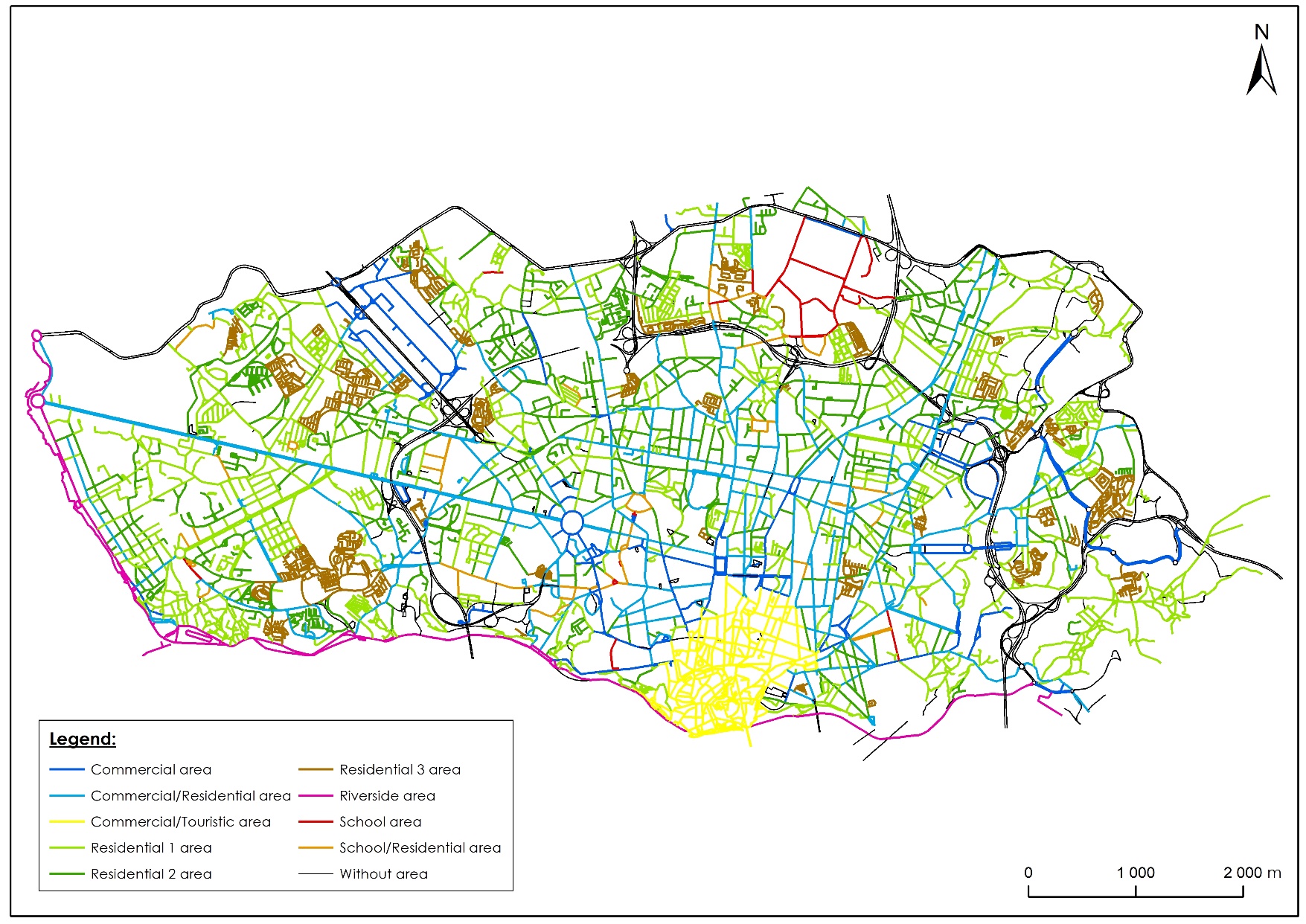


Figure SM2 - Porto city map, divided in the nine city areas


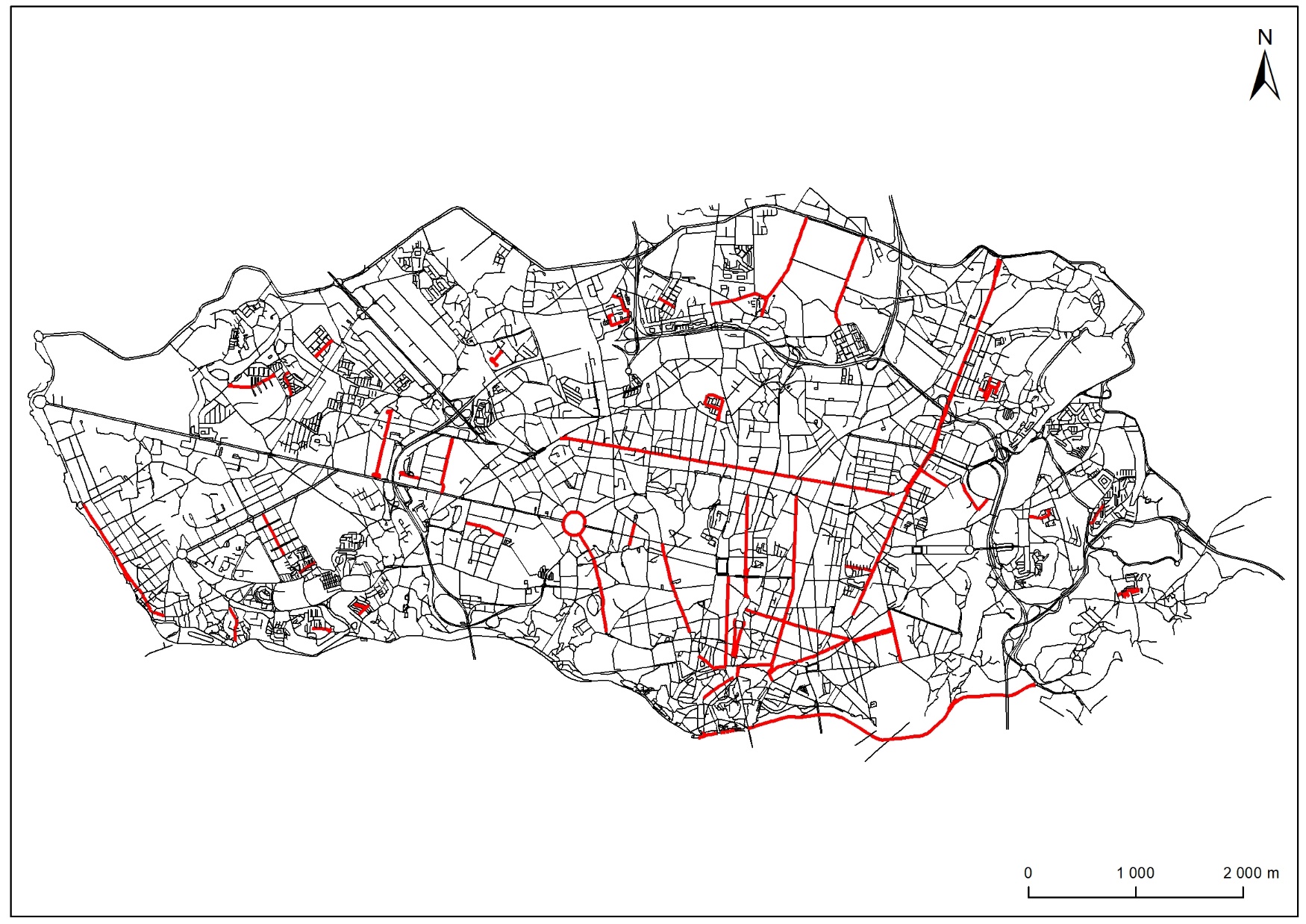


Figure SM3 – Porto city map with the quantified streets marked in red

Figure SM4 – Survey answers obtained for Question 3 for Porto city, divided by gender

Table SM1 - Waste categories, description, and criteria used for size classification

| **Waste category** | **Acronym** | **Description** | **Size** | | |
| --- | --- | --- | --- | --- | --- |
|  |  |  | **Small** | **Medium** | **Large** |
| Food Packaging | FP | Paper and plastic packages (ex. Water bottles, yogurts/groceries packaging) | ≥ 1-Euro coin;  < cigarette pack | ≥ cigarette pack;  < A4 sheet | ≥ A4 sheet |
| Non-Food Packaging | NFP | Paper and plastic packages (ex. Bus tickets, newspapers, cigarette packs, receipts) |  |  |  |
| Glass and glass pieces | GGP | All types of glass bottles, whole or broken | Glass piece | Bottles from 25 cL to 50 cL | Bottles from 50 cL to 1 L |
| Cigarette butts | CB | NA | 1 unit | NA | NA |
| Animal droppings | AD | NA | 1 unit | NA | NA |
| Mixed-waste bags | MWB | NA | 1 unit | NA | NA |
| Leaves and branches | LB | NA | Leaves | Small branches | Large branches |
| Incrustations | I | Ex. Oil or paint stains, chewing gums, bird dropping stains, human/animal urination stains | Chewing gums | Area < 500 cm^2^ | Area ≥ 500 cm^2^ |
| Organic food waste | OW | Ex. Apple pits, banana or orange peels, puke | ≥ 1-Euro coin;  < banana peel | ≥ banana peel;  < 20 L waste bag | ≥ 20 L waste bag |
| Other types of organic waste | OOW | Ex. Napkins, toilet paper, condoms, sanitary pads, syringes |  |  |  |
| Metals | M | Ex. Soda cans, metal bottle caps, random metal pieces | All metals excluding soda cans | NA | Soda cans |

NA - Not applicable.

Note: For the waste that does not belong to any of these 11 categories, the operator must allocate it to the category and size that seems more suitable.

Table SM2 – Total linear length (km) of each city area, with the acronym, and the respective percentage

| **City Area** | **Acronym** | **Length (km)** | **%** |
| --- | --- | --- | --- |
| **Commercial/Touristic Area** | **CT** | 37 | 6 |
| **Commercial Area** | **C** | 44 | 7 |
| **Residential 1 Area (single-family buildings)** | **R1** | 186 | 30 |
| **Residential 2 Area (multi-family buildings)** | **R2** | 132 | 21 |
| **Residential 3 Area (social-housing)** | **R3** | 83 | 13 |
| **Commercial/Residential Area** | **CR** | 95 | 15 |
| **School Area** | **S** | 7 | 1 |
| **School/Residential Area** | **SR** | 16 | 3 |
| **Riverside Area** | **Riv** | 19 | 3 |
| **Total** | | 619 | 100 |

Note: The total value shown of 619 km excludes the length of the streets classified as “Without area”, such as highways and their entries, or closed streets. Those represent 137 km of the total street length of Porto city (757 km).

Table SM3 – Factors (p_i j_) to standardise sizes depending on the waste category

| **Waste category** | **Acronym** | **Small (j=1)** | **Medium (j=2)** | **Large (j=3)** |
| --- | --- | --- | --- | --- |
| **Food packaging** | FP | 1 | 3 | 9 |
| **Non-food packaging** | NFP | 1 | 3 | 9 |
| **Organic food waste** | OW | 1 | 3 | 9 |
| **Other types of organic waste** | OOW | 1 | 3 | 9 |
| **Cigarette buts** | CB | 1 | - | - |
| **Animal droppings** | AD | 1 | - | - |
| **Mixed waste bags** | MW | 1 | - | - |
| **Leaves and branches** | LB | 1 | 1.5 | 2 |
|  |  |  |  |  |
|  | **Acronym** | **Chewing gums** | **Small**  **(< 500 cm^2^)** | **Large**  **(≥ 500 cm^2^)** |
| **Incrustations** | I | 0.5 | 0.8 | 1 |
|  |  |  |  |  |
|  | **Acronym** | **Glass pieces** | **Bottles**  **25 cL to 50 cL** | **Bottles**  **50 cL to 1 L** |
| **Glass and glass pieces** | GGP | 1 | 6 | 9 |
|  |  |  |  |  |
|  | **Acronym** | **Soda cans** | **Other metals** |  |
| **Metals** | M | 6 | 1 |  |

Note: For the Leaves and Branches category, small is for leaves, medium is for small branches, and large is for large branches.

Table SM4 – Porto streets analysed during the study

| **Nº** | **Street name** | **Google Maps link** |
| --- | --- | --- |
| **Commercial/Touristic Area** | | |
| **1** | Rua dos Clérigos | https://goo.gl/maps/AF2GxkS3gZ2zTX2o9 |
| **2** | Rua de Santa Catarina | https://goo.gl/maps/FwN6WKXM6csoMgZv9 |
| **3** | Rua de Fernandes Tomás | https://goo.gl/maps/cH6oYKByXF6f5Xhp7 |
| **4** | Rua de 31 de Janeiro e Praça da Batalha | https://goo.gl/maps/8GARnhLY7SL1hqcL8 https://goo.gl/maps/SxGTj1qi1g6Tf3CCA |
| **5** | Rua das Flores e Praça de Almeida Garrett | https://goo.gl/maps/uS44sAUYKk2pL3os8  https://goo.gl/maps/5jeELFhddiyH3pMv6 |
| **6** | Rua de Sá da Bandeira | https://goo.gl/maps/w36v6oSiG6WYng5o7 |
| **7** | Avenida dos Aliados | https://goo.gl/maps/jPxrXZAHp89ZTd5g7 |
| **8** | Rua do Almada | https://goo.gl/maps/MFW91SdzLoQ4CgT36 |
| **9** | Rua das Carmelitas | https://goo.gl/maps/5dBn3366QqnQP9fB8 |
| **10** | Zona Ribeira: Largo do Terreiro, Cais da Estiva e Cais da Ribeira | https://goo.gl/maps/S1oyFvZfcuvArUkK7  https://goo.gl/maps/K3SqBHVxGSYMYcdDA  https://goo.gl/maps/u5VsJTPaohZnYrMx7 |
| **Commercial Area** | | |
| **1** | Rua da Senhora da Luz | https://goo.gl/maps/NtSkJBUJj1hyixfw6 |
| **2** | Rua de Cedofeita | https://goo.gl/maps/nt82bNa1GfejqSBN9 |
| **3** | Rua de Júlio Dinis | https://goo.gl/maps/hQniePrnmQYdUkF69 |
| **4** | Rua de Santo Ildefonso | https://goo.gl/maps/5oQA7zSvCaTfn6nU7 |
| **5** | Praça de Mouzinho de Albuquerque (Rotunda da Boavista) | https://goo.gl/maps/Vjtcb85GXxsrE2Bj9 |
| **Residential 1 Area** | | |
| **1** | Rua do Monte Aventino | https://goo.gl/maps/v7cYDdXRBgDrNxPp8 |
| **2** | Rua de Garcia de Orta, Rua do Padre Luís de Almeida e Rua do Padre Fernão Cardim | https://goo.gl/maps/TUWz8VcMKguRyjLZ6  https://goo.gl/maps/gZ5M5sADf44Fy6tb9  https://goo.gl/maps/pAunoBHcDQUFvAJ96 |
| **3** | Rua das Dálias | https://goo.gl/maps/bSQS3SDCwt6857BF7 |
| **4** | Rua da Beneditina | https://goo.gl/maps/BmzthryiqhPX63Zd8 |
| **5** | Rua de Cascais e Rua de Miramar | https://goo.gl/maps/XbseXfmcfZmeG2en9  https://goo.gl/maps/SG5s3iWR39e5x32e7 |
| **6** | Rua de Feliciano de Castilho | https://goo.gl/maps/G7GB9JgsZEGa9Qox9 |
| **7** | Rua do Furriel Guilherme Dantas | https://goo.gl/maps/Bo7TbTK481YfpFN49 |

Table SM4 – Porto streets analysed during the study (continuation)

| **Nº** | **Street name** | **Google Maps link** |
| --- | --- | --- |
| **Residential 2 Area** | | |
| **1** | Rua de Robert Auzelle | https://goo.gl/maps/ixNcnKT77gCPThXT6 |
| **2** | Rua de Fernando Pessoa | https://goo.gl/maps/3Mo8N2EkriMztkjy6 |
| **3** | Rua de José Monteiro da Costa e Rua da Sociedade Protectora dos Animais | https://goo.gl/maps/dGe8JAyKrU7MLZ6D8  https://goo.gl/maps/Vkok1fVTGQCDZtD87 |
| **4** | Rua do Professor Agostinho da Silva | https://goo.gl/maps/JDCX9vAa6WaeVkMf8 |
| **5** | Rua de Adelaide Estrada | https://goo.gl/maps/q6HtVq2sqjymJ3b97 |
| **6** | Rua de João Baptista Lavanha | https://goo.gl/maps/Qq8LtXKc2rNKPKKLA |
| **Residential 3 Area** | | |
| **1** | Rua de Arzila | https://goo.gl/maps/KjWGNWFXUyboQAgb8 |
| **2** | Rua do Dr. Carlos Passos | https://goo.gl/maps/uqXvZcXrn6KZTdLFA |
| **3** | Rua do Engenheiro Pedro Inácio Lopes | https://goo.gl/maps/EWU535iPeNKbHF8R6 |
| **4** | Rua Manuel Pinheiro da Rocha | https://goo.gl/maps/TWEJdVJJWvx9i6AH7 |
| **5** | Rua de Diogo Macedo | https://goo.gl/maps/dTWcuRyDcoGvJJ4s9 |
| **6** | Rua da Guiné | https://goo.gl/maps/7gVnzbDKbKYpYPL17 |
| **7** | Rua de Nicolau Coelho | https://goo.gl/maps/Lzz1AYnCg6MHDbSZ6 |
| **8** | Rua de Camilo Pessanha | https://goo.gl/maps/8M6AEMz8EDGVBFtc7 |
| **Commercial/Residential Area** | | |
| **1** | Rua de S. João de Brito | https://goo.gl/maps/zXA2HaJReYvg4n457 |
| **2** | Rua de Luís de Aguiar | https://goo.gl/maps/4QMUisDzQCUtKC3k6 |
| **3** | Rua de Camões | https://goo.gl/maps/SoXTuXPHcan8k7QM8 |
| **4** | Rua da Constituição | https://goo.gl/maps/15H6weCvBahvYtsb9 |
| **5** | Avenida de Fernão de Magalhães | https://goo.gl/maps/AP2FkgEHz1uN7Mgt5 |
| **School Area** | | |
| **1** | Rua do Dr. António Bernardino de Almeida | https://goo.gl/maps/uGiR64B8G4g3zM4bA |
| **2** | Rua de António Carneiro | https://goo.gl/maps/5ZKhCcK3SvcsMrCG7 |
| **3** | Rua do Dr. Roberto Frias | https://goo.gl/maps/iMX7AX9g6R1tAe486 |
| **4** | Rua do Dr. Carlos Ramos | https://goo.gl/maps/sqHEazAVWXiaXGG38 |

Table SM4 – Porto streets analysed during the study (continuation)

| **Nº** | **Street name** | **Google Maps link** |
| --- | --- | --- |
| **School/Residential Area** | | |
| **1** | Rua de O Primeiro de Janeiro | https://goo.gl/maps/yUpJMTQfHLupHujG8 |
| **2** | Avenida de Camilo | https://goo.gl/maps/Vw15phM5hD1iirYu6 |
| **3** | Rua de Augusto Luso | https://goo.gl/maps/6f2zJyqDE1kmzx3LA |
| **4** | Rua do Conde de Avranches | https://goo.gl/maps/G5sCSBcaS1Zd4bf6A |
| **5** | Rua de Carlos Malheiro Dias | https://goo.gl/maps/tk5vBxozkuaqVBDQ9 |
| **Riverside Area** | | |
| **1** | Avenida do Brasil | https://goo.gl/maps/yYB6hqBwuBau8sFC7 |
| **2** | Avenida de Gustavo Eiffel e Avenida de Paiva Couceiro | https://goo.gl/maps/nth6qJ5NzcNZW8vA9  https://goo.gl/maps/PLnFo77en9hZe13M9 |

Table SM5 – Limits and linear adjustments for each category (i), for a 100 m^2^ area and small units of waste

| **Category (i)** | **Acronym** | **Minimum value** | **Maximum value** | **Linear adjustment (valid between maximum and minimum values)** |
| --- | --- | --- | --- | --- |
| **Food packaging** | FP | 3 | 22 | *CL*i = -5.3*R*i + 115.8 |
| **Non-Food packaging** | NFP | 5 | 30 | *CL*i = -4*R*i + 120 |
| **Glass and glass pieces** | GGP | 10 | 60 | *CL*i = -2*R*i + 120 |
| **Cigarette buts** | CB | 40 | 300 | *CL*i = -0.4*R*i + 115.4 |
| **Animal droppings** | AD | 0 | 2 | *CL*i = -50*Ri* + 100 |
| **Mixed waste bags** | MW | 0 | 2 | *CL*i = -50*R*i + 100 |
| **Leaves and branches** | LB | 80 | 200 | *CL*i = -0.8*R*i + 166.7 |
| **Incrustations** | I | 100 | 900 | *CL*i = -0.1*R*i + 112.5 |
| **Organic food waste** | OW | 1 | 6 | *CL*i = -20*R*i + 120 |
| **Other types of organic waste** | OOW | 1 | 4 | *CL*i = -33.3*R*i + 133.3 |
| **Metals** | M | 5 | 18 | *CL*i = -7.7*R*i + 138.6 |

Note 1: The application of the linear adjustment to the minimum or maximum levels could lead to values slightly different 100.0 or 0.0, due to rounding. Note 2: The limits for Leaves and Branches categories are for the sampling period of the study, so it does not consider the leaf falling period. To replicate this methodology during this period, those limits should be readjusted.

Table SM6 - Annoyance index for each waste category, w_i_, for sidewalks, gardens and tree grates/flowerpots

| **Category (i)** | **Acronym** | ***w_i_* for sidewalks** | ***w_i_* for gardens, tree grates/flowerpots** |
| --- | --- | --- | --- |
| **Food packaging** | FP | 0.092 | 0.107 |
| **Non-food packaging** | NFP | 0.083 | 0.096 |
| **Glass and glass pieces** | GGP | 0.105 | 0.122 |
| **Cigarette butts** | CB | 0.085 | 0.099 |
| **Animal droppings** | AD | 0.114 | 0.131 |
| **Mixed waste bags** | MW | 0.099 | 0.115 |
| **Leaves and branches** | LB | 0.060 | 0 |
| **Incrustations** | I | 0.078 | 0 |
| **Organic food waste** | OW | 0.097 | 0.113 |
| **Other types of organic waste** | OOW | 0.094 | 0.109 |
| **Metals** | M | 0.093 | 0.108 |

Note: There are two ranges of values because Leaves and Branches and Incrustations were not counted during the sample analysis on tree grates/flowerpots and gardens. So, the remaining parameters were readjusted.

Table SM7 – Weighting factors for each disposal place

| **Factor (x)** | **SW** | **TF** | **GR** | **GU** | **LT** |
| --- | --- | --- | --- | --- | --- |
| ***P*_x_** | 0.229 | 0.150 | 0.192 | 0.150 | 0.279 |

Note: SW – Sidewalks; TF – Tree grates/Flowerpots; GR – Gardens; GU – Gullies; LT – Litter bins

Table SM8 – Comprehensive summary table of all indicators with their definitions, formulas and units

| **Variables** | **Equations** | **Description** | **Units** |
| --- | --- | --- | --- |
| *p_ij_* | *-* | Size factor for each waste category (i) and size (j). | - |
| *r_ij_* | *-* | Units of waste found by category (i) and size (j). | - |
| *R_i_* | $R_{i}=\frac{\sum_{j=1}^{3} \left( p_{\mathrm{ij}}\times r_{\mathrm{ij}} \right)}{A}\times100$ | Equation to obtain the waste equivalent unit (Ri). (Note: The quantification results for the analysed samples of 250 or 500 m^2^ should be firstly standardised to 100 m^2^ and consider the smaller size of waste so that all results can be comparable.) | Nr. / 100 m^2^ |
| *CL_i_* | *-* | Cleanliness level for each waste category (i), obtained by different linear adjustments for each waste category (i), for a 100 m^2^ area and small units of waste. | % |
| *w_i_* | *-* | Annoyance index for each waste category (i). | - |
| *CL_x_* | *CL*_x_ = $\prod_{i=1}^{11} ({CL}_{i}^{w_{i}})$ | Equation to obtain the indicator value for each waste disposal place (x): sidewalks (CLSW), gardens (CLGR) and tree grates/flowerpots (CLTF). | % |
| *CL_GU_* | *CL*_GU_ = $\frac{N_{GU clean}}{N_{GU total}}$ × 100 | Equation to obtain the indicator value for gullies (CLGU).  (Note: This indicator is the ratio between the number of clean gullies and the total number of gullies analysed in that sample. Before the calculation, these numbers should be standardised to the number of gullies, clean and total, per 100 m^2^.) | % |
| *CL_LT_* | *CL*_LT_ = $\frac{N_{LT empty}}{N_{LT total}}$ × 100 | Equation to obtain the indicator value for litter bins (CLLT).  (Note: This indicator is the ratio between the number of empty litter bins (< 50% capacity) and the total number of litter bins analysed in that sample. Before the calculation, these numbers should be standardised to the number of litter bins, empty and total, per 100 m^2^.) | % |
| *P_x_* | *-* | Weighting factor for each waste disposal place (x). | - |
| *CL* | *CL* = $\frac{\sum({CL}_{x} \times P_{x})}{\sum P_{x}}$ | Equation to obtain the cleanliness level (CL) of a sample, street, area, city, etc. | % |

Table SM9 – *CL_i_* distribution for Sidewalks (SW), Tree grates/Flowerpots (TF) and Gardens (GR), for all the city areas

| **Graphic *CL_i_* distribution** | **Area** |
| --- | --- |
|  | **Commercial/Touristic**   - In SW, I and CB are in large amount, but this do not affect CL because these categories have small w_i_ values. - For NFP, even though they are not in great amount, they have a higher w_i_ value, so CL is affected. - In TF, the biggest problem is for OW category, due to the amount found at *Rua Santa Catarina.* |
|  | **Commercial**   - The biggest cleaning problems are for NFP category at SW. - There are AD issues at TF and GR, however, as not a lot of samples were analysed with these disposal places, no significant conclusions can be drawn. - CB are also a problem at SW in this area (typical fact verified in commercial areas). |
|  | **Commercial/Residential**   - AD category affects CL value (typical fact verified in residential areas), mainly at TF (because there were analysed only a few samples with GR). - NFP category affects the 3 disposal places analysed, more evidently at SW. - Note for residential areas^[[1]](#footnote-2)^: CL value decreases with the increase of people influx. This means R1 areas are cleaner than R2 areas which in turn are cleaner than CR areas, generally for all the categories. |

Table SM9 - *CL_i_* distribution for Sidewalks (SW), Tree grates/Flowerpots (TF) and Gardens (GR), for all the city areas *(continuation)*

| **Graphic *CL_i_* distribution** | **Area** |
| --- | --- |
|  | **Residential 1**   - At SW, even though LB category has a low w_i_ value, CL_LB_ is 0%, so this means LB are a cleaning problem in these areas. - AD category is also a cleaning problem, at the three disposal places analysed, more pronounced at GR, with CL_AD_ values of about 40%. |
|  | **Residential 2**   - LB are also a cleaning problem at SW, with CL_LB_ values close to 0%. - NFP category affects the three disposal places analysed, more evidently at SW. - AD category is clearly a cleaning problem in these areas, more pronounced at TF and GR (CL_AD_ values of 0%). |
|  | **Residential 3**   - FP and NFP categories are a cleansing problem for the three disposal places analysed (CL_i_ values of 0% for SW and GR). - AD category affects CL values in these areas, more pronounced at GR with CL_AD_ values of about 40%. - CL values are also affected by CB amount at SW (CL_CB_ of about 60%). |

Table SM9 - *CL_i_* distribution for Sidewalks (SW), Tree grates/Flowerpots (TF) and Gardens (GR), for all the city areas *(continuation)*

| **Graphic *CL_i_* distribution** | **Area** |
| --- | --- |
|  | **School**   - AD category is a cleaning problem mainly for GR (there were analysed a lot of samples with this disposal place, which validate this situation). - LB category affects cleaning level in these areas for SW, because these are areas with a lot of trees. - NFP category also affects CL values, more evidently at GR and SW. |
|  | **School/Residential**   - CL_i_ values for NFP category are lower than the ones for scholar areas, for the three disposal places analysed, more pronounced at GR (0%) and SW (20%). - AD category is a cleaning problem at TF and GR, both with CL_i_ values of 0%. |
|  | **Riverside**   - Generally, this is not an area that represents a cleaning problem. - However, there were found AD at the GR and TF analysed, but the samples quantified were not in great amount so any significant conclusions can be drawn. |

Table SM9 - *CL_i_* distribution for Sidewalks (SW), Tree grates/Flowerpots (TF) and Gardens (GR), for all the city areas *(continuation)*

| **Graphic *CL_i_* distribution** | **Area** |
| --- | --- |
|  | **Porto city**   - Clearly, AD and NFP categories are problems for the streets cleaning levels. - These categories were found, more frequently, at SW and GR for NFP, and at TF and GR for AD. - Even though not so evidently, CB and I are also a cleaning problem at SW. |

Label: FP – Food Packaging, NFP – Non-food Packaging, GGP – Glass and Glass Pieces, CB – Cigarette Butts, AD – Animal Droppings, MW – Mixed-waste bags, LB – Leaves and Branches, I – Incrustations, OW – Organic Waste, OOW – Other type of organic waste, M – Metals, SW – Sidewalks, TF – Tree grates/Flowerpots, GR – Gardens.

Note: The Incrustations and Leaves and Branches categories are not quantified on tree grates/flowerpots nor in gardens, so *CL_i_* values for these categories were not obtained. As it so, these values in the graphics above are all 100%, since do not represent a cleaning problem.

Table SM10 – CL_x_ distribution for Sidewalks (SW), Tree grates/Flowerpots (TF), Gardens (GR), Gullies (GU) and Litter Bins (LB), for all the city areas

| **Graphic *CL_x_* distribution** | **Area** |
| --- | --- |
|  | **Commercial/Touristic**   - Cleanliness levels are high for the all the disposal places, except for LT, that are a bit full (70%). - Only a few samples with GR were analysed (almost 0%), so any significant conclusions can be drawn for CL_GR_ values. |
|  | **Commercial**   - Cleanliness problems are in GU, with CL_GU_ of 60%. - CL_x_ values for TF and GR are high, however, only a few samples with these locals were analysed, so any significant conclusions can be drawn. |
|  | **Residential 1**   - There are cleanliness problems on the SW (30%) due to the Leaves and Branches found (section 3.1). - The great amount of waste from this category found, CL_GU_ value is compromised due to clogging (60%). - Only a few samples with GR were analysed, so CL_GR_ values are not conclusive. |

Table SM10 – CL_x_ distribution for Sidewalks (SW), Tree grates/Flowerpots (TF), Gardens (GR), Gullies (GU) and Litter Bins (LB), for all the city areas (continuation)

| **Graphic *CL_x_* distribution** | **Area** |
| --- | --- |
|  | **Residential 2**   - There are cleanliness problems on every analysed place, everyone with CL_x_ values lower than 40%. - The huge amount of LB found (section 3.1), compromises both CL_SW_ and CL_GU_ values. - CL_TF_ and CL_GR_ values are compromised by AD (section 3.1), however there were not analysed a lot of samples with TF. - The LT analysed were almost always full. |
|  | **Residential 3**   - The main cleanliness problems are on SW and GR (CL_x_ values lower than 40%). - There were analysed a lot of samples with GR (more than 80%), so CL_GR_ values is reliable. - The amount of LT analysed is low and they are not frequently used, so CL_LT_ value is high (close to 100%). |
|  | **Commercial/Residential**   - The cleanliness problem is at TF, due to the AD found (CL_TF_ of 40%). - At SW, NFP is the category that mainly affect CL_SW_ value. - Only a few samples with GR were analysed, typical fact of commercial areas. |

Table SM10 – CL_x_ distribution for Sidewalks (SW), Tree grates/Flowerpots (TF), Gardens (GR), Gullies (GU) and Litter Bins (LB), for all the city areas (continuation)

| **Graphic *CL_x_* distribution** | **Area** |
| --- | --- |
|  | **School**   - There are cleanliness problems in the majority of the places, except on TF. - However, only a few samples were analysed with GR and LT. This means that more samples need to be analysed so concrete conclusions can be drawn about the CL_x_ values for these two places. - As for residential areas, LB and AD are the categories that mainly affect CL_x_ values. |
|  | **School/Residential**   - In this area, the cleanliness problems found in residential areas and in scholar areas get together. - LB category affect CL_SW_ and CL_GU_ values. - AD category affect CL_SW_, CL_TF_ and CL_GR_ values. However, only about 20% of the analysed samples have GR, so the CL_GR_ value requires a carefully analysis. |
|  | **Riverside**   - There were not found big cleanliness problems, so CL_x_ values are all close to 100%. - Only a few samples have TF and GR, however the ones analysed were super clean. |

Table SM10 – CL_x_ distribution for Sidewalks (SW), Tree grates/Flowerpots (TF), Gardens (GR), Gullies (GU) and Litter Bins (LB), for all the city areas (continuation)

| **Graphic *CL_x_* distribution** | **Area** |
| --- | --- |
|  | **Porto city**   - CL values are all about 50%, except for LT with almost 80%. So, this is the cleanest place analysed. - The most problematic place is SW, mainly due to the amount of AD found at residential and scholar areas. |

Label: SW – Sidewalks, TF – Tree grates/Flowerpots, GR – Gardens, GU – Gullies, LT – Litter bins

Table SM11 – *P_x_* values: original and the ones obtained from adjustments

|  | **P_SW_** | **P_TF_** | **P_GR_** | **P_GU_** | **P_LB_** |
| --- | --- | --- | --- | --- | --- |
| **Original** | **0,229** | **0,150** | **0,192** | **0,150** | **0,279** |
| **Adjustment 1 (5%)** | 0,212 | 0,153 | 0,196 | 0,153 | 0,285 |
| **Adjustment 2 (10%)** | 0,196 | 0,157 | 0,200 | 0,157 | 0,291 |
| **Adjustment 3 (20%)** | 0,165 | 0,162 | 0,208 | 0,162 | 0,302 |
| **Adjustment 4 (40%)** | 0,121 | 0,154 | 0,237 | 0,143 | 0,345 |
| **Adjustment 5 (80%)** | 0,047 | 0,129 | 0,228 | 0,080 | 0,515 |
| **Adjustment 6 (99%)** | 0,046 | 0,115 | 0,220 | 0,087 | 0,532 |

Label: SW – Sidewalks, TF – Tree grates/Flowerpots, GR – Gardens, GU – Gullies, LB – Litter Bins.

Table SM12 – *w_i_* values: original and the ones obtained from adjustments

|  | **Sidewalk** | | | | | | |
| --- | --- | --- | --- | --- | --- | --- | --- |
|  | **Original** | **5%** | **10%** | **20%** | **40%** | **80%** | **99%** |
| **FP** | **0,092** | 0,095 | 0,098 | 0,104 | 0,114 | 0,151 | 0,181 |
| **NFP** | **0,083** | 0,078 | 0,072 | 0,062 | 0,044 | 0,015 | 0,001 |
| **GGP** | **0,105** | 0,109 | 0,112 | 0,119 | 0,131 | 0,172 | 0,207 |
| **CB** | **0,085** | 0,079 | 0,074 | 0,064 | 0,045 | 0,015 | 0,001 |
| **AD** | **0,114** | 0,107 | 0,099 | 0,086 | 0,061 | 0,021 | 0,001 |
| **MW** | **0,099** | 0,102 | 0,106 | 0,112 | 0,123 | 0,162 | 0,195 |
| **LB** | **0,060** | 0,056 | 0,052 | 0,045 | 0,032 | 0,011 | 0,001 |
| **I** | **0,078** | 0,081 | 0,083 | 0,088 | 0,097 | 0,128 | 0,154 |
| **OW** | **0,097** | 0,100 | 0,103 | 0,109 | 0,121 | 0,018 | 0,001 |
| **OOW** | **0,094** | 0,097 | 0,100 | 0,106 | 0,117 | 0,154 | 0,185 |
| **M** | **0,093** | 0,096 | 0,099 | 0,105 | 0,116 | 0,153 | 0,074 |
|  | **Tree grates/Flowerpots and Gardens** | | | | | | |
|  | **Original** | **5%** | **10%** | **20%** | **40%** | **80%** | **99%** |
| **FP** | **0,107** | 0,109 | 0,112 | 0,116 | 0,123 | 0,139 | 0,153 |
| **NFP** | **0,096** | 0,089 | 0,082 | 0,069 | 0,047 | 0,103 | 0,110 |
| **GGP** | **0,122** | 0,125 | 0,127 | 0,132 | 0,140 | 0,159 | 0,175 |
| **CB** | **0,099** | 0,101 | 0,103 | 0,107 | 0,114 | 0,129 | 0,142 |
| **AD** | **0,131** | 0,121 | 0,112 | 0,094 | 0,065 | 0,019 | 0,001 |
| **MW** | **0,115** | 0,118 | 0,120 | 0,124 | 0,132 | 0,149 | 0,164 |
| **LB** | **-** | - | - | - | - | - | - |
| **I** | **-** | - | - | - | - | - | - |
| **OW** | **0,113** | 0,115 | 0,118 | 0,122 | 0,130 | 0,147 | 0,162 |
| **OOW** | **0,109** | 0,111 | 0,114 | 0,118 | 0,125 | 0,016 | 0,001 |
| **M** | **0,108** | 0,110 | 0,113 | 0,117 | 0,124 | 0,140 | 0,093 |

Label: FP – Food Packing, NFP – Non-Food Packaging, GGP – Glass and Glass Pieces, CB – Cigarette Butts, AD – Animal Droppings, MW – Mixed-waste bags, LB – Leaves and Branches, I – Incrustations, OW – Organic food waste, OOW – Other types of organic waste, M - Metals

1. R3 areas were excluded from this reasoning because they are atypical situations – social housing. [↑](#footnote-ref-2)
